# Effect of Supplemental Oxygen on von Willebrand Factor Activity and Ristocetin Cofactor Activity in Patients at Risk for Cardiovascular Complications Undergoing Moderate-to High-Risk Major Noncardiac Surgery—A Secondary Analysis of a Randomized Trial

**DOI:** 10.3390/jcm12031222

**Published:** 2023-02-03

**Authors:** Katharina Horvath, Alexander Taschner, Nikolas Adamowitsch, Markus Falkner von Sonnenburg, Edith Fleischmann, Barbara Kabon, Melanie Fraunschiel, Christian Reiterer, Alexandra Graf

**Affiliations:** 1 Department of Anaesthesia, General Intensive Care Medicine and Pain Medicine, Medical University of Vienna, 1090 Vienna, Austria; 2 IT Systems and Communications, Medical University of Vienna, 1090 Vienna, Austria; 3 Center for Medical Data Science, Medical University of Vienna, 1090 Vienna, Austria

**Keywords:** von Willebrand factor, ristocetin, endothelial demage, noncardiac surgery

## Abstract

Increased von Willebrand Factor (vWF) activity mediates platelet adhesion and might be a contributor to the development of thrombotic complications after surgery. Although in vitro studies have shown that hyperoxia induces endovascular damage, the effect of perioperative supplemental oxygen as a possible trigger for increased vWF activity has not been investigated yet. We tested our primary hypothesis that the perioperative administration of 80% oxygen concentration increases postoperative vWF activity as compared to 30% oxygen concentration in patients at risk of cardiovascular complications undergoing major noncardiac surgery. A total of 260 patients were randomly assigned to receive 80% versus 30% oxygen throughout surgery and for two hours postoperatively. We assessed vWF activity and Ristocetin cofactor activity in all patients shortly before the induction of anesthesia, within two hours after surgery and on the first and third postoperative day. Patient characteristics were similar in both groups. We found no significant difference in vWF activity in the overall perioperative time course between both randomization groups. We observed significantly increased vWF activity in the overall study population throughout the postoperative time course. Perioperative supplemental oxygen showed no significant effect on postoperative vWF and Ristocetin cofactor activity in cardiac risk patients undergoing major noncardiac surgery. In conclusion, we found no significant influence of supplemental oxygen in patients undergoing major non-cardiac surgery on postoperative vWF activity and Ristocetin cofactor activity.

## 1. Introduction

Major surgery is an independent risk factor for the development of postoperative thromboembolic events [1]. It has been shown that surgical trauma, anesthesia, intraoperative hemodynamic and fluid perturbations, and perioperative inflammation are important causes vascular endothelial damage [2]. This is an important fact, since the activation of endothelial cells by damage leads to the expression of adhesion molecules including P-selectin, E-selectin, and vWF [3,4]. Von Willebrand Factor (vWF) is a large multimeric glycoprotein and a key component in hemostasis [5,6]. VWF is synthesized in megakaryocytes and endothelial cells and stored in Weibel–Palade bodies [7]. It is known that vWF is a strong mediator for platelet adhesion, aggregation and thrombus formation, and more importantly, increases significantly after surgery [8,9]. Therefore, increased vWF activity caused by activated endothelial cells might be one cause for the increased incidence of postoperative thromboembolic events.

Previous studies indicated that hyperoxia induces endovascular damage [10,11,12]. In detail, it has been shown in in vitro studies that an oxygen concentration of 95% is associated with the occurrence of DNA damage of endothelial cells and fibroblasts [12]. Although it is becoming more evident that supplemental oxygen has no effect on wound healing or cardiovascular complications, a strong consensus about the most beneficial concentration does still not exist [13,14,15]. Subsequently, intraoperative administered oxygen concentration is still varying and mainly dependent on the attending anesthesiologists [16]. In this context, possible effects of perioperative supplemental oxygen on the integrity of endothelial cells are clinically relevant; however, data from the perioperative setting are still lacking.

Thus, we tested in this pre-planned secondary analysis of a prospective randomized clinical trial the hypothesis that perioperative administration of 80% oxygen concentration increases postoperative vWF activity as compared to 30% oxygen concentration in patients at risk of cardiovascular complications undergoing major noncardiac surgery. We further evaluated if supplemental oxygen increases postoperative Ristocetin cofactor activity.

## 2. Materials and Methods

This is a pre-planned secondary analysis of a double-blinded randomized clinical trial that investigated the effect of supplemental oxygen on postoperative maximum N-terminal pro brain natriuretic peptide (NT-proBNP) concentrations in patients at risk for cardiovascular complications undergoing major noncardiac surgery [17]. The study was conducted at the Medical University of Vienna according to the Declaration of Helsinki and Good Clinical Practice. The study was approved by the local Institutional Review Board and was registered at ClinicalTrials.gov (Registration number: NCT 03388957; Principal Investigator: Prof. Dr. Edith Fleischmann; Date of registration: 2 December 2017) and at the European Trial Database (EudraCT 2017-003714-68). This study was approved by the University’s Ethics Committee (Ethikkomission Medizinische Universität Wien; Borschkegasse 8b/6, 1090, Vienna, Austria; EK-Number 1744/2017; Chairperson Prof. Martin Brunner) on 13 November 2017. We obtained written informed consent from all patients before randomization. Inclusion and exclusion criteria and a detailed description of our study protocol and the randomization procedure were published previously [17].

We recorded demographic data including age, sex, BMI, American Society of Anesthesiologists (ASA) physical status, comorbidities, long-term medication, type of surgery, ABO blood type, and preoperative laboratory values from all patients. We further recorded duration of anesthesia and surgery, fluid and anesthesia management, and intra- and postoperative blood pressure. Blinded research personnel drew all study specific pre- and postoperative blood samples. We assessed vWF activity, Ristocetin cofactor activity, and static oxidation-reduction potential (sORP) in all patients shortly before induction of anesthesia, within two hours after surgery and on the first and third postoperative day. We further measured ADAMTS13 (a disintegrin and metalloproteinase with a thrombospondin type 1 motif, member 13), an enzyme that cleaves vWF, before surgery in all patients.

Blinded research personnel obtained all data. All data were recorded and stored in the data management system ‘Clincase’(v2.7.0.12, Berlin, Germany) hosted by IT Systems & Communications, Medical University of Vienna, Vienna, Austria.

We included all patients who were enrolled into the main trial for this secondary analysis. The study was originally planned for the main study outcome, the maximum BNP value over the first 3 days. We re-estimated the sample size for this secondary analysis based on previous results on vWF to get an evaluation of the available sample size. It was shown previously that adverse cardiac events after noncardiac surgery were associated with postoperative vWF activity of 150% ± 60% compared with postoperative maximum vWF activity of 125% ± 50% in patients without postoperative cardiac events [18]. Therefore, based on the aforementioned study, we assumed an absolute difference of 17% in postoperative vWF activity as clinically meaningful. Using the given assumptions, a two-sided t-test, we calculated that at least 123 patients per group are needed to detect a significant difference between both groups at a significance level of 0.05 with 90% power. Thus, the given sample size of 260 patients (130 patients per group) may be adequately powered.

Descriptive statistics (mean, standard deviation and quantiles) for vWF activity and Ristocetin cofactor activity were calculated separately for each time point and the 80% and 30% oxygen group. To investigate the difference in time course of vWF activity and Ristocetin cofactor activity between both randomization groups, first linear regression models for vWF activity and Ristocetin cofactor activity were performed accounting for time, group and the interaction between time and group as fixed factor and patient as random factor. Further, we used univariable linear regression models (with random factor patient) for the possible influence factors including time as well as the baseline covariates age, BMI, sex, ASA, history of coronary artery disease, peripheral artery disease, stroke, heart failure, diabetes, hypertension, type of procedure (open versus laparoscopic), type of surgery, ABO blood type, and preoperative ADAMTS13 and blood loss on perioperative vWF and Ristocetin cofactor activity. All factors being significant (with a *p* < 0.05) in the simple models where then included in a multivariable regression model (with random factor patient). To evaluate a possible correlation between the perioperative trend of vWF activity and oxidative stress—assessed via sORP measurements—we performed a linear regression model for vWF as the dependent variable, accounting for time, sORP and the interaction between time and sORP as well as patient as a random factor. All *p*-values < 0.05 were considered statistically significant. We used R.4.2.2 (SAS Institute, Cary, NC, USA) and SAS 9.4 (SAS Institute, Cary, NC, USA) for statistical analysis.

## 3. Results

We present the analysis of 258 patients who were enrolled in our main trial between December 2017 and December 2019 at the Medical University of Vienna. A total of 130 patients were randomly assigned to receive 80% oxygen throughout surgery and for two hours postoperatively, and 130 patients were randomly assigned to receive 30% oxygen throughout surgery and for two hours postoperatively. Two patients in the 80% group were excluded from analysis because surgery was postponed. Thus, overall, 258 patients were analyzed. Baseline characteristics as well as intra- and postoperative characteristics were published previously and did not differ between the groups [19].

### 3.1. Primary Outcome

**Figure 1:**
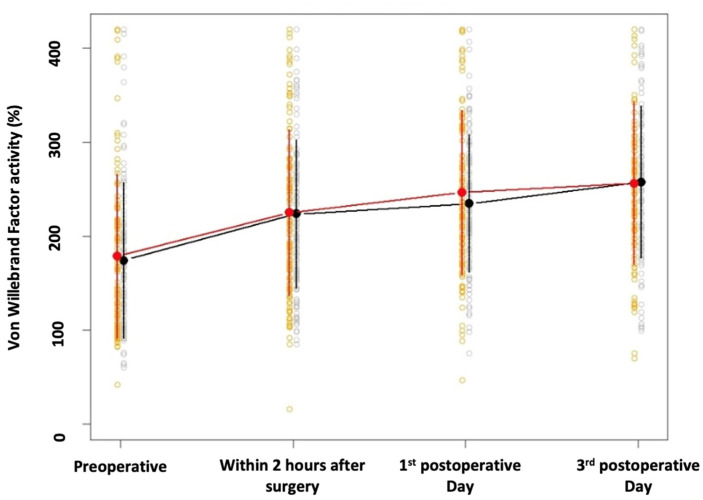
Time course for von Willebrand Factor activity. Mean values (dots) and standard deviations (vertical lines) for vWF separately for time and the 30%-group (black) as well as the 80%-group (red). The blank dots give the values of the observed individuals, separately for the two groups.

Descriptive statistics of vWF activity separately for each randomization group and time are shown in Table 1. The perioperative trends of vWF activity for both study groups are shown in Figure 1. We found no significant difference in vWF activity in the overall perioperative time course between the 80% and the 30% oxygen groups (estimated effect: 0.297; 95% CI −4.154 to 4.749; *p* = 0.896). Furthermore, a significant difference between the 80% and 30% oxygen group was found at no time point (Table 1).

We observed significantly increased vWF activity in the overall study population over time (*p* < 0.001) as compared to baseline. vWF activity increased on average by 19.264 (95% CI 17.040 to 21.488) per day.

### 3.2. Secondary Outcome: Ristocetin Cofactor Activity

Descriptive statistics of Ristocetin cofactor activity separately for randomization group and time are shown in Table 1.

We found no significant difference in Ristocetin cofactor activity in the overall perioperative time course between the 80% and the 30% oxygen groups (estimated effect: 1.003; 95% CI 20.500 to 32.528; *p* = 0.818). Furthermore, a significant difference between the 80% and 30% oxygen group was found at no time point (Table 1).

We observed significantly higher Ristocetin cofactor activity in the overall study population over time (*p* < 0.001) as compared to baseline. On average, Ristocetin cofactor activity increased by 26.969 (95% CI 22.954 to 30.984) per day.

### 3.3. Analyses of Possible Confounding Factors

#### 3.3.1. Von Willebrand Factor Activity

Significantly higher postoperative vWF activities were observed for females as compared to males (*p* = 0.044) for patients with a history of peripheral artery disease (*p* = 0.036), patients with a blood type other than O (*p* < 0.001), and patients with pancreatic surgery (*p* < 0.001) in the univariable model. Patients undergoing renal surgery (*p* = 0.002) or prostatectomy (*p* = 0.012) had significantly smaller vWF activity. Age, BMI, ASA, history of coronary artery disease, stroke, heart failure, diabetes, hypertension or preoperative ADAMTS13 or blood loss did not show evidence for an association with vWF activity. For all time points, patients with laparoscopic surgery showed lower postoperative vWF activity as compared to open procedures (*p* < 0.001) (Table 2).

The factors time point, type of surgery, sex, history of peripheral artery disease, blood type, pancreatic or renal surgery as well as prostatectomy were included in the multivariable model. All parameters except renal surgery and prostatectomy remained significant in the multivariable model (Table 3).

#### 3.3.2. Ristocetin Cofactor Activity

Significantly higher postoperative Ristocetin cofactor activity was found for increasing age (*p =* 0.019), females as compared to males (*p =* 0.027), patients with peripheral artery disease (*p =* 0.001), patients without history of hypertension (*p* = 0.001), patients with blood type other than O (*p* < 0.001) and patients having pancreatic surgery (*p* < 0.001) in the univariable model. Significantly lower Ristocetin cofactor activity was found in patients having renal surgery (*p =* 0.003). BMI, ASA, history of coronary artery disease, stroke, heart failure, diabetes or preoperative ADAMTS13 or blood loss did not show evidence for an association with Ristocetin cofactor activity. For all time points, patients with laparoscopic surgeries showed lower postoperative Ristocetin cofactor activity (*p* < 0.001) (Supplemental Materials, Table S1: Univariable regression model Ristocetin). The factors time point, type of surgery, age, sex, history of peripheral artery disease, history of hypertension, blood type, pancreatic, and renal surgery were included in the multivariable model. All parameters except for pancreatic or renal surgery remained significant in the multivariable model (Supplemental Materials, Table S2: Multivariable regression model Ristocetin).

#### 3.3.3. vWF Activity and SORP

Over the perioperative time course, a significant positive correlation between the trend of vWF activity and sORP was observed in the overall study population (estimated effect: 0.380; 95% CI 0.170 to 0.590; *p* < 0.001).

## 4. Discussion

In this secondary analysis, we assessed endothelial damage via consecutive vWF activity measurements. We observed no significant effect of perioperative 80% oxygen concentration on postoperative vWF activity as compared to perioperative 30% oxygen concentration. Furthermore, we did not observe a significant difference in postoperative Ristocetin cofactor activity between the two groups.

In the original trial, we showed that the administration of supplemental oxygen was not associated with significant changes in postoperative maximum NT-proBNP and Troponin T concentrations [19]. Previous secondary analyses of this trial showed that supplemental oxygen was also not associated with significant changes in postoperative catecholamine levels [20] as well as postoperative Copeptin or oxidative stress levels [21,22].

A previous trial showed no adverse effects of supplemental oxygen on the incidence of myocardial injury after noncardiac surgery (MINS) in patients with cardiovascular risk factors undergoing major noncardiac surgery [23]. In another trial that evaluated the effect of supplemental oxygen in surgical site infections, the authors did not detect any significant effect of supplemental oxygen [14]. A post hoc analysis of this trial also showed that supplemental oxygen did not increase overall postoperative mortality [24]. Therefore, it seems likely that supplemental oxygen does not significantly affect the development of postoperative complications after major noncardiac surgery.

Surgery leads to postoperative inflammation, stress, and hypercoagulation [18,25,26,27,28]. We observed a significant increase in vWF activity after surgery in the overall patient population independent of the administered oxygen concentration. Supplemental oxygen is associated with increased inflammatory response, specifically in alveolar epithelium and in human cardiac myocytes [29,30]. Pure oxygen causes increased reactive oxygen stress leading to inflammation and ultimately to alveolar cell death [29]. In contrast to these findings, we observed that vWF activity was independent of the administered oxygen concentration. This is also true regarding oxidative stress. In a previous sub-analysis, we showed that the increase in oxidative stress did not differ significantly between the 80% and the 30% oxygen group [22]. Based on the current evidence, we are convinced that surgical trauma, anesthesia, fluid, and hemodynamic perturbations are the predominant factors for endothelial dysfunction rather than the administration of higher oxygen concentrations. To evaluate if oxidative stress might have affected vWF activity, we also performed a post hoc correlation. We found that postoperative oxidative stress correlates significantly with postoperative vWF activity. This further confirms that surgical trauma might be the most reasonable cause for increased oxidative stress and might therefore be the most important trigger factor for postoperative vascular damage represented by our increased vWF activity.

Nearly all of our patients underwent surgery for cancer. It is known that vWF activity is increased in cancer patients, which might explain the higher incidence of coagulopathies in these patients [31]. Moreover, even the type of cancer plays a significant role [32]. Specifically, patients with pancreatic cancer have a high risk of developing thromboembolic events [32]. This is consistent with our observations. We observed significantly higher vWF activity in patients with pancreatic cancer. Therefore, it seems reasonable that vWF activity might be an important contributor for coagulopathies, specifically in patients undergoing surgery for pancreatic cancer.

Peripheral artery disease is strongly associated with atherosclerosis, vascular damage, hypercoagulability, and an increased incidence of thromboembolic events [33]. We found significantly higher vWF and Ristocetin cofactor activity in patients with peripheral artery disease. A possible explanation might be that peripheral artery disease is associated with endothelial dysfunction, especially in the perioperative period, where endothelial dysfunction is caused by surgical trauma [34,35,36]. Since vWF is a potent clotting factor, it might very well be that vWF plays an important role in the postoperative pathogenesis of thromboembolic events.

Interestingly, in contrast to vWF activity, we observed significantly higher Ristocetin cofactor activity in hypertensive patients. It is known that hypertension is associated with vascular injury [37] which might have resulted in higher Ristocetin cofactor activity due to vascular damage.

Plasma levels of vWF are approximately 25% higher in patients with blood type A, B, or AB as compared to blood type O [38]. While the molecular mechanisms for these differences have not been entirely clarified, it is of high clinical importance, as the risk of venous as well as arterial thromboembolic events is significantly higher in patients with blood type A, B, or AB [39,40]. Patients presenting with blood type AB are shown to have the highest rate of venous thrombotic events as compared to blood group O, followed by B and A [41]. Several other studies also linked the incidence of myocardial infarction (MI) and coronary artery disease (CAD) to the ABO blood type, where group O has the lowest risk for MI and CAD [42,43].

This study has some limitations. This was a pre-planned secondary analysis. The study was powered to detect the effect between 80% versus 30% perioperative oxygen concentration on postoperative maximum NT-proBNP concentration [19]. We only included patients undergoing major noncardiac cancer surgery. Therefore, we were not able to compare perioperative vWF activity between patients undergoing cancer versus non-cancer surgery, which might have helped to underline the high risk of postoperative thromboembolic events in patients having cancer surgery. Our rate of thromboembolic events was far too small to evaluate the association between postoperative vWF activity and the incidence of thromboembolic events. In fact, only four patients developed pulmonary embolism within 30 days after surgery. Therefore, the clinical impact of our results needs to be evaluated in a large observational study with adequate power to detect a higher number of postoperative thromboembolic events. Further it is well known that endothelial damage is associated with an increase in various biomarkers. In detail, endothelial damage leads to an increase in fibrinogen [44]. Additionally, excess endothelial stimulation in patients with peripheral artery disease is associated with D-Dimer and Thrombin-Antithrombin III levels [44]. Lastly, in our study, patients in the non-intervention group received a FiO_2_ of 0.3, which is still higher than the physiological level of 0.21. Hafner et al. showed in an in vitro study that even slight increases in the oxygen concentration are associated with increases in VEGF secretion [30]. Therefore, it cannot be ruled out that oxygen might have affected vWF activity in our non-intervention group. However, the use of 21% oxygen during surgery is relatively uncommon; thus, 30% oxygen might better reflect current clinical practice.

## 5. Conclusions

In conclusion, we found no significant influence of supplemental oxygen in patients undergoing major non-cardiac surgery on postoperative vWF activity and Ristocetin cofactor activity.

## Figures and Tables

**Table 1:** Descriptive statistics for vWF activity and Ristocetin cofactor activity. VWF activity and Ristocetin cofactor activity at each timepoint are presented as median (25th quartile; 75th quartile). All *p*-values are for two-tailed Mann–Whitney U tests.

	80% Oxygen		30% Oxygen		*p*-Value
vWF activity, %					
Baseline	156.5	[112.75; 212.5]	155.5	[119; 200.75]	0.78
2 h postoperative	211	[160; 274.5]	214	[163; 266]	0.85
Postoperative day 1	228	[180; 315.75]	219.5	[186; 279.5]	0.45
Postoperative day 3	244	[199.5; 302]	256	[193; 317]	0.82
Ristocetin cofactor activity, %					
Baseline	142.5	[113.75; 208.75]	152	[116.5; 199.5]	0.88
2 h postoperative	227.5	[164.25; 306.25]	247	[198; 315]	0.19
Postoperative day 1	255.5	[190; 359.75]	245	[201; 286]	0.56
Postoperative day 3	247.5	[199.25; 311]	270	[203.5; 340]	0.36

**Table 2:** Univariable regression models vWF. The estimated effect sizes, confidence levels (CL) and *p*-values were calculated using univariable regression models. pre, preoperative; 2 h post, within two hours after surgery; POD, postoperative day; BMI, body mass index; ASA, American Society of Anesthesiologists.

Variable	Comparison	Effect	Lower CL	Upper CL	*p*-Value
Time	Overall Trend Test	19.264	17.040	21.488	<0.001
	pre vs. 2 h post	−46.672	−55.050	−38.295	<0.001
	pre vs. POD 1	−62.434	−70.973	−53.894	<0.001
	pre vs. POD 3	−80.874	−89.720	−72.028	<0.001
Time × Group	Overall Trend Test	0.297	−4.154	4.749	0.896
	Group 30% vs. 80% pre	−2.964	−23.547	17.619	0.777
	Group 30% vs. 80% 2 h post	0.099	−20.528	20.726	0.993
	Group 30% vs. 80% POD 1	−11.570	−32.475	9.335	0.277
	Group 30% vs. 80% POD 3	0.507	−20.852	21.866	0.963
	Group 30% pre vs. 2 h post	−48.180	−59.995	−36.365	<0.001
	Group 30% pre vs. POD 1	−57.891	−70.157	−45.625	<0.001
	Group 30% pre vs. POD 3	−82.617	−95.187	−70.048	<0.001
	Group 80% pre vs. 2 h post	−45.117	−57.009	−33.224	<0.001
	Group 80% pre vs. POD 1	−66.497	−78.401	−54.584	<0.001
	Group 80%: pre vs. POD 3	−79.146	−91.610	−66.682	<0.001
Type of surgery	Laparoscopic vs. Open	−48.264	−66.232	−30.297	<0.001
Time × Type of surgery	Overall Trend Test	1.523	−3.171	6.216	0.524
Liver	Yes vs. No	10.534	−19.790	40.858	0.495
Colorectal	Yes vs. No	−11.296	−31.163	13.330	0.431
Pancreatic	Yes vs. No	55.257	29.981	80.534	<0.001
Renal	Yes vs. No	−38.300	−62.435	−14.166	0.002
Prostatectomy	Yes vs. No	−36.031	−64.046	−8.017	0.012
Cystectomy	Yes vs. No	−17.482	−48.574	13.610	0.269
Gynecological	Yes vs. No	27.488	−19.689	74.660	0.252
Other	Yes vs. No	3.228	−26.021	32.477	0.828
Age		1.059	−0.134	2.252	0.082
BMI		0.301	−1.552	2.154	0.750
Sex	Female vs. Male	19.584	0.561	38.607	0.044
ASA	3,4 vs. 1,2	15.168	−4.580	34.916	0.132
History of Coronary Artery Disease	Yes vs. No	2.912	−18.077	23.901	0.785
History of Peripheral Artery Disease	Yes vs. No	26.660	1.698	51.622	0.036
History of stroke	Yes vs. No	−2.287	−34.760	30.186	0.890
History of Heart failure	Yes vs. No	8.693	−26.490	43.875	0.627
Diabetes	Yes vs. No	−0.345	−20.530	19.839	0.973
History of Hypertension	Yes vs. No	−26.494	−61.876	8.889	0.142
Blood type	0 vs. A,B,AB	51.242	32.961	69.523	<0.001
pre ADAMTS		0.002	−0.100	0.105	0.962
Type of surgery	Laparoscopic vs. Open	−48.264	−66.232	−30.297	<0.001
Blood Loss		0.007	−0.007	0.021	0.345

**Table 3:** Multivariable regression model vWF. The estimated effect sizes, confidence levels and p-values were calculated using multivariable regression models (with random factor patient). pre, preoperative; 2 h post, within two hours after surgery; POD, postoperative day; CL, confidence level.

Variable	Comparison	Effect	Lower CL	Upper CL	*p*-Value
Time	pre vs. 2 h post	−45.736	−54.239	−37.233	<0.001
	pre vs. POD 1	−61.587	−70.243	−52.931	<0.001
	pre vs. POD 3	−80.248	−80.305	−89.272	<0.001
Type of surgery	Laparoscopic vs. Open	−30.367	−50.177	−10.557	0.003
Sex	Female vs. Male	18.570	1.194	35.946	0.036
History of Peripheral Artery Disease	Yes vs. No	26.305	4.018	48.592	0.021
Blood type	0 vs. A,B,AB	55.529	38.568	72.489	<0.001
Pancreatic	Yes vs. No	28.625	5.024	52.225	0.018
Renal	Yes vs. No	−16.297	−41.125	8.531	0.197
Prostatectomy	Yes vs. No	−25.026	−52.736	2.685	0.077

## Data Availability

The datasets analyzed during the current study are available from the corresponding author on reasonable request.

## Supplementary Materials

The following supporting information can be downloaded at: https://www.mdpi.com/article/10.3390/jcm12031222/s1, Table S1: Univariable regression model Ristocetin, Table S2: Multivariable regression model Ristocetin.
