# Supplementary material for: Effect of Supplemental Oxygen on von Willebrand Factor Activity and Ristocetin Cofactor Activity in Patients at Risk for Cardiovascular Complications Undergoing Moderate-to High-Risk Major Noncardiac Surgery—A Secondary Analysis of a Randomized Trial
Source: J Clin Med. 2023 Feb 3;12(3):1222. doi: 10.3390/jcm12031222 (PMC9918071; doi:10.3390/jcm12031222)
Supplement: Supplementary file 1 [file jcm-12-01222-s001.zip › jcm-2172132-supplementary.pdf]

Supplementary Online Content

Table S1. Univariable regression model Ristocetin
Table S2. Multivariable regression model Ristocetin

This supplementary material has been provided by the authors to give readers additional information about their work.

Table S1: Univariable regression model Ristocetin

Variable	Comparison	Effect	lower CL	upper CL	*p*-value
Time	Overall Trend Test	26.969	22.954	30.984	<0.001
	pre vs. post 2h	-85.794	-99.489	-72.100	<0.001
	pre vs. POD 1	-97.980	-112.800	-83.157	<0.001
	pre vs. POD 3	-110.390	-125.590	-95.185	<0.001
Time x Group	Overall Trend Test	1.003	20.500	32.528	0.818
	Group 30% vs. 80% pre	-7.009	-32.518	18.500	0.590
	Group 30% vs. 80% 2h post	14.243	-13.215	41.700	0.309
	Group 30% vs. 80% POD 1	-21.596	-51.396	8.205	0.155
	Group 30% vs. 80% POD 3	-21.260	-51.465	8.946	0.167
	Group 30% pre vs. 2h post	-96.485	-115.860	-77.105	<0.001
	Group 30% pre vs. POD 1	-90.103	-111.470	-68.733	<0.001
	Group 30% pre vs. POD 3	-116.940	-138.670	-95.222	<0.001
	Group 80% pre vs. 2h post	-75.233	-94.494	-55.972	<0.001
	Group 80% pre vs. POD 1	-104.690	-125.180	-84.203	<0.001
	Group 80% pre vs. POD 3	-104.350	-125.540	-83.163	<0.001
Type of surgery	Laparoscopic vs. Open	-46.450	-68.040	-24.860	<0.001
Time x Type of surgery	Overall Trend Test	-1.643	-10.117	6.832	0.704
Liver	Yes vs. No	7.732	-26.191	41.654	0.654
Colorectal	Yes vs. No	1.187	-24.375	26.750	0.927
Pancreatic	Yes vs. No	50.437	22.120	78.755	<0.001
Renal	Yes vs. No	-42.086	-69.177	-14.995	0.003
Prostatectomy	Yes vs. No	-25.053	-56.165	6.060	0.114
Cystectomy	Yes vs. No	5.792	-29.658	41.241	0.748

Gynaecological	Yes vs No.	44.646	-7.929	97.221	0.096
Other	Yes vs No.	-9.827	-43.576	23.923	0.567
Age		1.674	0.279	3.068	0.019
BMI		-0.260	-2.440	1.919	0.814
Sex	Female vs. Male	25.548	2.931	48.164	0.027
ASA	3,4 vs. 1,2	20.331	-2.855	43.517	0.085
History of Coronary Artery Disease	Yes vs. No	0.2205	-24.918	25.359	0.986
History of Peripheral Artery Disease	Yes vs. No	47.752	19.162	76.342	0.001
History of stroke	Yes vs. No	12.031	-27.374	51.435	0.548
History of Heart failure	Yes vs. No	-12.321	-54.938	30.296	0.570
Diabetes	Yes vs. No	-1.291	-25.354	22.773	0.916
History of Hypertension	Yes vs. No	-67.306	-106.880	-27.732	0.001
Blood type	0 vs A, B, AB	46.111	23.804	68.418	<0.001
pre ADAMTS		-0.024	-0.141	0.092	0.679

Table S1. The estimated effect sizes, confidence levels (CL) and *p*-values were calculated using univariable regression models. pre, preoperative; 2 h post, within two hours after surgery; POD, postoperative day

Table S2: Multivariable regression model Ristocetin

Variable	Comparison	Effect	lower CL	upper CL	p-value
Time	pre vs. 2h post	-85.774	-99.513	-72.035	<0.001
	pre vs. POD 1	-95.391	-110.220	-80.559	<0.001
	pre vs. POD 3	-108.000	-123.240	-92.771	<0.001
Type of surgery	Laparoscopic vs Open	-38.570	-60.868	-16.273	<0.001
Age		2.087	0.808	3.366	0.002
Sex	Female vs. Male	28.671	8.148	49.194	0.006
History of Peripheral Artery Disease	Yes vs. No	39.979	13.043	66.915	0.004
History of Hypertension	Yes vs. No	-64.034	-102.010	-26.056	0.001
Blood type	0 vs A, B, AB	43.726	23.585	63.867	<0.001
Pancreatic surgery	Yes vs. No	19.136	-8.436	46.708	0.173
Renal surgery	Yes vs. No	-10.750	-38.968	17.469	0.454

Table S2. The estimated effect sizes, confidence levels (CL) and *p*-values were calculated using multivariable regression models (with random factor patient). pre, preoperative; 2 h post, within two hours after surgery; POD, postoperative day
